# RNA-binding protein signaling in adult neurogenesis

**DOI:** 10.3389/fcell.2022.982549

**Published:** 2022-09-16

**Authors:** Jackie Ngai-Man Chan, Dalinda Isabel Sánchez-Vidaña, Shailendra Anoopkumar-Dukie, Yue Li, Lau Benson Wui-Man

**Affiliations:** ^1^ Department of Rehabilitation Sciences, The Hong Kong Polytechnic University, Hong Kong, Hong Kong SAR, China; ^2^ Mental Health Research Centre, The Hong Kong Polytechnic University, Hong Kong, Hong Kong SAR, China; ^3^ School of Pharmacy and Medical Sciences, Griffith University, Gold Coast, QLD, Australia; ^4^ State Key Laboratory of Component-Based Chinese Medicine, Institute of Traditional Chinese Medicine, Tianjin University of Traditional Chinese Medicine, Tianjin, China

**Keywords:** adult neurogenesis, neurogenesis regulation, miRNA, RNA-binding proteins, gene regulaiton

## Abstract

The process of neurogenesis in the brain, including cell proliferation, differentiation, survival, and maturation, results in the formation of new functional neurons. During embryonic development, neurogenesis is crucial to produce neurons to establish the nervous system, but the process persists in certain brain regions during adulthood. In adult neurogenesis, the production of new neurons in the hippocampus is accomplished via the division of neural stem cells. Neurogenesis is regulated by multiple factors, including gene expression at a temporal scale and post-transcriptional modifications. RNA-binding Proteins (RBPs) are known as proteins that bind to either double- or single-stranded RNA in cells and form ribonucleoprotein complexes. The involvement of RBPs in neurogenesis is crucial for modulating gene expression changes and posttranscriptional processes. Since neurogenesis affects learning and memory, RBPs are closely associated with cognitive functions and emotions. However, the pathways of each RBP in adult neurogenesis remain elusive and not clear. In this review, we specifically summarize the involvement of several RBPs in adult neurogenesis, including CPEB3, FXR2, FMRP, HuR, HuD, Lin28, Msi1, Sam68, Stau1, Smaug2, and SOX2. To understand the role of these RBPs in neurogenesis, including cell proliferation, differentiation, survival, and maturation as well as posttranscriptional gene expression, we discussed the protein family, structure, expression, functional domain, and region of action. Therefore, this narrative review aims to provide a comprehensive overview of the RBPs, their function, and their role in the process of adult neurogenesis as well as to identify possible research directions on RBPs and neurogenesis.

## Introduction

Neurogenesis is the formation process of new neurons derived from neural progenitor cells (NPCs), occurring in several regions of the brain including the olfactory epithelium, hippocampus, and subventricular zone (SVZ) (Kempermann et al., 2004). It consists of four stages namely cell proliferation, migration, differentiation, and integration into the existing circuit (Ming and Song, 2011). In adulthood, neurogenesis takes place mainly in two regions of the brain, the SVZ and subgranular zone (SGZ) of the dentate gyrus in the hippocampus (Sanai et al., 2004; Curtis et al., 2007; Semple et al., 2013; Sánchez-Vidaña et al., 2019). Hippocampal neurogenesis is a pivotal physiological process involved in the regulation of cognitive and emotional behaviors such as the formation of spatial memory in learning, the response to stress, and mood (Yau et al., 2011; Chan et al., 2017; Fung et al., 2021). Various gene modulatory pathways participate in the neuronal growth process which consists of neuronal relocation, neuronal plasticity, synaptic formations, and dendritic and axonal outgrowth (Mills and Janitz, 2012; Qu et al., 2020). The dynamic modulation of alternative splicing (AS) in the nervous system is essential for the orchestrated regulation of protein-protein interactions, transcription systems, and neuronal growth (Qu et al., 2020).

More than 500 RNA-binding proteins (RBPs) have been identified in the human genome (Vogel and Richard, 2012). RBPs are in charge of complex RNA-protein and protein-protein interactions to regulate RNA metabolism (Vogel and Richard, 2012). Each RBP interacts with RNA with different affinities (Vogel and Richard, 2012). Gene expression is regulated by a variety of proteins, but RBPs represent a distinct subgroup within these proteins (Gerstberger et al., 2014). RBPs are responsible for regulating gene expression in various ways, including splicing, cleavage, polyadenylation, RNA stabilization, RNA localization, RNA editing, and translation. Several genetic processes, e.g., AS and the utilization of poly(A) sites mediated by the neuro-oncological ventral antigen (NOVA) protein, an RBPs first identified in autoimmune motor neuron diseases, involve RBPs (Licatalosi et al., 2008; Eom et al., 2013; Tang et al., 2020). Malfunctioning RBPs are associated with genetic and somatic disorders, for instance neurodegenerative, autoimmune, and cancer diseases (Lukong et al., 2008). As post-transcriptional steps are usually carried out in membrane- and phase-separated subcellular compartments, RBPs’ regulatory functions are also impacted by their subcellular localization.

Apart from RBPs, micro RNAs (miRNAs) are another common type of gene expression mediators. The regulation of gene expression by RBPs and miRNAs can take place in an antagonistic fashion in which RBPs and miRNAs can act on the same targets or nearby regulatory elements (Velasco et al., 2019). For example, several miRNAs preferentially bind to Pumilio (PUM), a group of the PUF family of sequence-specific RNA-blinding proteins, and have binding motifs that complement the PUM recognition sites in reverse order (Shao et al., 2013). Upon binding, miRNA-binding efficiency increases due to PUM binding to transcripts, which in turn leads to an increase in shared target decay (Shao et al., 2013). Alternatively, they can inhibit the expression of a common target, a single transcript, which suggests that the interaction between RBPs and miRNAs takes place in a complex manner (Velasco et al., 2019). These regulators are involved in neurogenesis and brain development processes (Velasco et al., 2019). Therefore, changes at functional or gene expression levels caused by RBPs and miRNAs could contribute to neurological disorders and brain tumors (Velasco et al., 2019).

**FIGURE 1:**
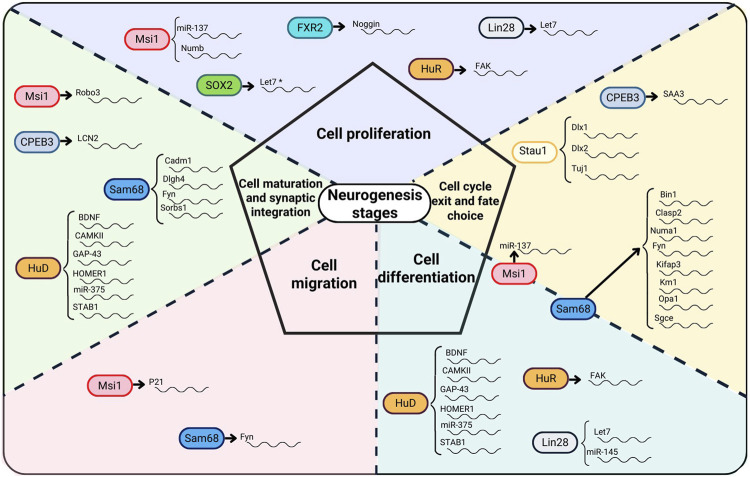
RBPs, their RNA targets, and the role of RBPs at different neurogenesis stages. RBPs acting on different stages of the neurogenesis process appear on the dotted lines of the neurogenesis stages that they regulate. In SOX2, * indicates an indirect interaction with let7 as SOX2 suppresses the expression of let7 expression by maintaining Lin28 expression.

**FIGURE 2:**
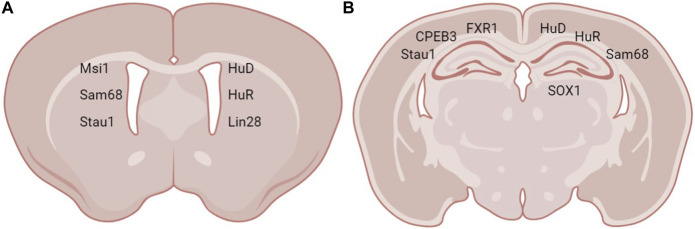
Expression sites of RBPs in neurogenic regions. RBPs expressed in **(A)** the SVZ and **(B)** the hippocampus.

The translation of numerous mRNAs in the brain are controlled by their interaction with ribonucleoprotein (RNP) granules, which are made up of translational machinery, core RBPs, and miRNAs (Kiebler and Bassell, 2006). RBPs regulate the trafficking of certain mRNAs into dendrites, bundle them into RNP granules, and may control the timing and location of their translation in response to the synaptic activity (Kiebler and Bassell, 2006). Due to these characteristics, RBPs are in a special position to regulate developmental processes by coordinating the translation of a group of functionally linked mRNAs (Moore, 2005; Keene, 2007). Neurons have the ability to adjust neuronal output and consolidate alterations in synaptic connections thanks to local protein synthesis (Bramham and Wells, 2007; Licatalosi et al., 2008). The moderation of nervous system architecture is subjected to a variety of spatio-temporal gene regulatory mechanisms, including control of protein synthesis *via* RBPs (Conlon and Manley, 2017; Qu et al., 2020). A comprehensive analysis of RBPs involved in neurogenesis, their structure, function, and RNA targets is presented below. A summary of the function of RBPs at the different stages of neurogenesis as well as the RBPs’ RNA targets and neurogenic regions are shown in Figure 1 and the expression of RBPs in neurogenic regions is illustrated in Figure 2.

## RNA-binding proteins involved in adult neurogenesis

### Cytoplasmic Polyadenylation Element binding proteins—3

The RNA-binding protein known as the Cytoplasmic Polyadenylation Element-Binding Protein (CPEB) family is essential for synaptic plasticity (Fernández-Miranda and Méndez, 2012). The CPEB family consists of four members (CPEB1, CPEB2, CPEB3, and CPEB4) that recognize the same Cytoplasmic Polyadenylation Element (CPE) found in the 3′untranslated region (3′UTR) of target mRNAs (Fernández-Miranda and Méndez, 2012; Parisi et al., 2021). CPEBs 2–4 are highly related, while CPEB1 is the most distant member (Wang et al., 2010; Fernández-Miranda and Méndez, 2012). These proteins are expressed in the brain (Theis et al., 2003), and they share a similar structure (Huang et al., 2006) with the carboxy-terminal region consisting of two RNA recognition motifs (RRMs) and two zinc-fingers (Hake et al., 1998). CPEB proteins (CPEBs) are capable of repressing or activating the translation of target mRNAs by shortening or stretching the poly-A tail (Wakiyama et al., 2000).

A recent discovery of three later members in this family (CPEB2-4) revealed the additional regulatory potential and biological functions of cytoplasmic polyadenylation (Mendez and Richter, 2001; Wang et al., 2010). By controlling the translation of many plasticity-related proteins (PRPs) RNAs in neurons, CPEBs limit the strength of glutamatergic synapses (Peng et al., 2010; Wang and Huang, 2012; Chao et al., 2013). CPEBs regulate the translation of mRNA that has been made inactive by closed-loop regulation. Translation is inhibited by a closed-loop structure between the 3′UTR and 5′UTR (Kang and Han, 2011).

In the process of establishing and maintaining neuronal development and synaptosomes, CPEB3 regulates translation by guiding necessary protein modifications (Qu et al., 2020). Therefore, CPEB3 serves as a mediator of translational activity in neurons of several identified mRNA targets (Ford et al., 2020). The function of CPEB3-related translation in neuronal development, migration, and synaptogenesis is modulated by several mechanisms (Peng et al., 2010; Hosoda et al., 2011). Genes related to transcription, neurodevelopment, and neurogenesis were enriched in CPEB3-bound genes. Several identified mRNA targets in neurons are translated by CPEB3. CPEB3 modulates the differential expression of genes associated with neurogenesis in HT22 cells, a mouse hippocampal cell line (Qu et al., 2020). CPEB3-regulated alternative splicing on control and CPEB3 overexpressing cells was examined using RNA-seq (Qu et al., 2020). By analyzing alternative splicing and differential gene expression, global CPEB3-RNA interaction has been elucidated using RNA-seq and iRIP-seq in neurons (Qu et al., 2020). In HT22 cells, CPEB3 had an insignificant impact on gene expression, involving 31 upregulated genes and 23 downregulated genes (Qu et al., 2020). Furthermore, overexpression of CPEB3 increased LCN2 mRNA levels in HT22 cells suggesting that CPEB3 modulates LCN2 pre-mRNA splicing (Qu et al., 2020). LCN2 is an alternative pathway for the delivery and uptake of physiological iron (Qu et al., 2020). The role of LCN2 in cell iron transport and homeostasis has recently been investigated (Devireddy et al., 2005; Bao et al., 2010). The LCN2 gene encodes an iron-binding protein that has been shown to regulate the density and morphology of hippocampal dendritic spines in the brain (Mucha et al., 2011). In addition, LCN2 plays a crucial role in neurogenesis, regulating NPCs maintenance, self-renewal, proliferation, differentiation as well as hippocampal plasticity (Mucha et al., 2011). Out of the four CPEBs, CPEB3 binds to LCN2 more efficiently (Qu et al., 2020). CPEB3 overexpression (CPEB3-OE) cell lines had an elevated level of SAA3, an acute-phase protein with cytokine-like properties. As a key modulator of neuronal survival and death, SAA3 is critical during inflammation (Huang et al., 2006). These findings contribute to the existing knowledge on the mechanisms that modulate neurogenesis and neuronal development mediated by CPEBs such as CPEB3 (Qu et al., 2020).

### Fragile X-related proteins (Fragile X-related protein 1, FXR1)

The family Fragile X-Related Proteins (FXRs) includes the Fragile X Mental Retardation Protein (FMRP), involved in a condition known as fragile X syndrome, and FMPR’s autosomal paralogs, the RBPs Fragile X-related protein 1 and 2 (Li and Zhao, 2014; Nishanth and Jha, 2022). FXRs are highly expressed in cortical neurons, cerebellar Purkinje neurons, and the brain stem (Patzlaff et al., 2018). FXRs are also expressed in dendrites, presynaptic spines, and axons in the thalamus, the CA3 region, and the olfactory bulb (Patzlaff et al., 2018). FXR2 is expressed in neuronal RNA granules containing FXRs and plays a critical role in the formation of such granules (Li and Zhao, 2014).

Structurally, FXR1 and FXR2 are very similar as they have two RNA-binding domains that are highly conserved, namely the polyribosome binding domain and the nuclear localization sequence (NLS) domain (Fernández et al., 2015; Patzlaff et al., 2017; Patzlaff et al., 2018). These proteins also contain an N-terminal protein-binding domain (NTD), which is responsible for the protein-protein interactions, two heterogeneous nuclear ribonucleoprotein (hnRNP) homology domains (KH), a nuclear export sequence (NES), and an arginine-glycine-glycine box (RGG) (Patzlaff et al., 2018). Although these proteins share some similar functional domains, they differ in the C termini region and the NLS domain (Li and Zhao, 2014). For instance, FXR2 contains an RG cluster in the C-terminal region which is not the case with FXR1 which contains an RGG box (Fernández et al., 2015). These structural differences explain their different RNA-binding properties (Fernández et al., 2015). The proteins of this family are homologous in their RNA-binding domains and can form hetero-multidimers. The heteromultimerization properties suggest that FXR1 and FXR2 have similar binding abilities to regulate protein translation (Patzlaff et al., 2018).

The members of this family can bind to RNA and associate with polyribosomes (Guo et al., 2011), and they can mediate RNA stability and translational efficiency (Patzlaff et al., 2018). One mechanism of action proposed for FXR2 is the recruitment of the translational machinery and therefore increase mRNA translation (Fernández et al., 2015). FXR2 regulates the circadian behavioral rhythms and plays a role in plasticity (Li and Zhao, 2014). FXR1 and FXR2 expression in the dentate gyrus is similar; however, from the functional point of view, these proteins play different roles (Patzlaff et al., 2017). FMRP, FX1, and FXR2 regulate adult neurogenesis having different functions (Guo et al., 2011; Li and Zhao, 2014; Patzlaff et al., 2017). For example, both FMRP and FXR2 suppress cell proliferation while FXR1 promotes it (Patzlaff et al., 2017). FXR2 is one of the several RBPs regarded as a critical regulator of neurogenesis (Nishanth and Jha, 2022).

FXR2 targets Noggin mRNA to regulate neurogenesis in the dentate gyrus of the adult brain (Nishanth and Jha, 2022). FXR2 inhibits Noggin protein expression by decreasing the stability of Noggin mRNA (Guo et al., 2011). Noggin’s role is to maintain cell pluripotency in stem cells (Guo et al., 2011). It also acts as a bone morphogenetic protein (BMP) inhibitor to trigger cell proliferation and neuronal differentiation (Guo et al., 2011). In the dentate gyrus NPCs lacking FXR2, the protein levels of Noggin increase, and the BMP pathway is blocked (Patzlaff et al., 2018).

In the dentate gyrus, the regulation of Noggin/BMP mediated signaling *via* FXR2 only takes place in this neurogenic region (Patzlaff et al., 2018) and FXR2 suppresses the expression levels of Noggin leading to an antagonistic effect on BMP signaling (Guo et al., 2011). On the contrary, this is not the case in the SVZ as FXR2 and Noggin are expressed in different cell types (Patzlaff et al., 2018). Consequently, the absence of FXR2 would not have any effect on the levels of the Noggin protein in the SVZ (Guo et al., 2011; Faigle and Song, 2013; Patzlaff et al., 2018). Considering that Noggin is found in the ependymal cells while FXR2 is expressed in neural progenitor cells, the regulation of Noggin expression by FXR2 is not direct (Guo et al., 2011). Noggin and FXR2 are colocalized in the dentate gyrus cells, and the lack of FXR2 in this type of cell results in increased cell proliferation of these cells (Guo et al., 2011). Furthermore, the lack of FXR2 results in decreased levels of PSD95 due to lower translational efficiency (Fernández et al., 2015). Low levels of FXR2 can affect stem cell proliferation and differentiation in the dentate gyrus of the hippocampus (Guo et al., 2011). However, this phenomenon is not observed in the SVZ (Guo et al., 2011; Faigle and Song, 2013).

### Hu antigen D and Hu antigen R

Hu antigen R (HuR), also known as HuA or embryonic lethal, abnormal vision-like 1 (ELAVL1), is an RBP member of the protein family embryonic lethal abnormal vision, or ELAV/Hu (Yao et al., 1993). The ELAV family also includes HuB, HuC, and HuD, the latter also known as ELAVL4 (Yao et al., 1993; McMahon and Ruggero, 2018). HuD is one of the earliest markers of neuronal lineage and is abundantly expressed in the mature nervous system (Bronicki and Jasmin, 2013) while HuR is ubiquitously expressed and translocated between the cytoplasm and the nucleus through the hinge region (Han et al., 2022). HuD, as well as other members of the ELAV family, is predominantly expressed in differentiated neurons (Dell’Orco et al., 2020). Expression of HuD is mainly restricted to particular neuronal populations such as the large pyramidal-like neurons in the layer of the neocortex and the Purkinje cells in the cerebellar cortex, the CA1-4 of the hippocampus, dorsal root ganglia, motor neurons in the spinal cord, mitral cells in the olfactory bulb, ganglion and internal plexiform layers in the retina, and neurons in the enteric nervous system (Bronicki and Jasmin, 2013). HuD is found in the mitral cells of the olfactory bulb, which receive afferent fibers from different cell types in the olfactory system and are crucial for the analysis of signals at the olfactory bulb level (Tepper et al., 2021). HuR is ubiquitously expressed (Dell’Orco et al., 2020). Within the cell, HuR is abundantly found in the cytoplasm with low expression in the nucleus ((Bronicki and Jasmin, 2013; Wang et al., 2019). In adult NPCs, HuR is found in neurogenic regions (Wang et al., 2019).

The members of the ELAV family have three RNA binding domains which are known as RNA recognition motifs (RRMs) (Burd and Dreyfuss, 1994). The RRMs are highly conserved in these proteins, but the hinge region located between the second and third RRM is different among them (Good, 1995). HuD and HuR bind to adenosine/uridine (A/U)-rich elements (AREs) (Fernández et al., 2015). Both proteins are involved in the regulation of different functions such as splicing, translation, and stability of several mRNAs (Fernández et al., 2015). The RRMs in ELAV proteins are the recognition sites that bind to specific target RNAs (Bronicki and Jasmin, 2013). ELAV proteins can form homo- and multimers on target mRNAs which indicates that this property has been evolutionarily conserved (Bronicki and Jasmin, 2013). For instance, HuD interacts with other proteins, including homo- and hetero-mutimerization with other ELAV proteins, *via* the third RRM (Bronicki and Jasmin, 2013). HuD stabilizes target mRNAs by binding to the 3′UTR by the first and second MMRs and the poly (A) tails by the third MMR (Dell’Orco et al., 2020). For HuR to act, it translocates from the nucleus into the cytoplasm where it regulates transcription stability and translation (Ghosh et al., 2009).

In the adult brain, HuD is involved in the regulation of neuronal plasticity, nerve injury, learning and memory, and neuronal diseases (Bronicki and Jasmin, 2013; Dell’Orco et al., 2020; Tepper et al., 2021). HuD functions as a post-translational regulator of mRNAs associated with neuronal differentiation and synaptic plasticity, including STAB1, GAP-43, BDNF, CAMKII, and HOMER1 as well as mechanisms of learning and memory (Dell’Orco et al., 2020; Tepper et al., 2021). For instance, an increase in HuD levels in the hippocampus has been found during dissociative- and special-learning and memory paradigms (Dell’Orco et al., 2020). HuD is known to regulate about 131 non-coding RNAs such as Y3 RNA which is found to interact with HuD, but its function remains unknown (McMahon and Ruggero, 2018). HuD also plays a role in the regulation of cell fate (Tebaldi et al., 2018).

HuD participates in different stages of neuronal differentiation and maturation processes, including neurogenesis, axonal and dendritic outgrowth, and cell remodeling (Abdelmohsen et al., 2010; Dell’Orco et al., 2020). Recently, HuD has been shown to be a key role player in adult neurogenesis (Wang et al., 2015), particularly in NSC differentiation into neuronal lineage. Mechanistically, HuD enhances the stability of the pre-mRNA of special AT-rich DNA-binding protein 1 (SATB1) by binding to the 3′UTR (Wang et al., 2015). SATB1 is an essential component for neuronal differentiation, and HuD deficiency would trigger a decrease in SATB1, which would suppress NSC differentiation (Wang et al., 2015). HuD promotes mRNA stability, and it also mediates the localization and translation of transcripts in neurites and the cytoplasm (Bronicki and Jasmin, 2013). For instance, GAP-43 and Tau are HuD target mRNAs that are involved in axonal outgrowth (Bronicki and Jasmin, 2013). HuD colocalizes GAP-43 and Tau during neuronal differentiation (Bronicki and Jasmin, 2013).

In the dentate gyrus of the hippocampus, HuD and its target mRNAs increase following neuro-toxin-induced injury (Bronicki and Jasmin, 2013). HuD has also been shown to localize to dendrite spines, and interact with mRNAs that encode synaptic proteins (Bronicki and Jasmin, 2013). These findings suggest that HuD is involved in neuronal plasticity mechanisms (Bronicki and Jasmin, 2013). In the hippocampus, higher binding of PSD95 mRNA and HuD takes place in the absence of FMRP (Fernández et al., 2015). FMRP, HuR, and HuD colocalize in different regions such as the neuronal cell bodies, dendritic processes in the CA3 region of the dentate gyrus, and primary neurons (Fernández et al., 2015). HuD acts by stabilizing PSD95 mRNA (Fernández et al., 2015). Both FMRP and HuD play an important role in the regulation of neuronal morphology, maturation, differentiation, and cytoskeletal organization (Fernández et al., 2015). Changes in the expression of HuD can affect mechanisms of special learning and memory processes in the hippocampus (Tepper et al., 2021).

HuD major effector is miR-375 which plays a role in neurite growth and dendritic maintenance (Abdelmohsen et al., 2010). Other HuD targets include those encoding for GAP-43, p21Waf1, and acetylcholinesterase, among others (Abdelmohsen et al., 2010). miR375 has an antagonistic effect on HuD as it suppresses its expression by destabilizing HuD mRNA which affects its translation (Abdelmohsen et al., 2010). Downregulation of HuD alters target genes involved in neuronal development, function, and neurite outgrowth (Abdelmohsen et al., 2010). This antagonistic regulatory effect of miR-375 on HuD affects translation processes that are essential for neurite growth, dendrite stability, and synapses as well as maintenance and plasticity of neuronal circuits (Abdelmohsen et al., 2010). For instance, the brain-derived neurotrophic factor (BDNF) is essential to maintaining neuronal mechanisms of plasticity, neuronal outgrowth, and neuronal differentiation (Abdelmohsen et al., 2010). Inhibition of BDNF mediated by miR-375 takes place as a result of the downregulation of HuD demonstrating that HuD can interact at posttranslational level with BDNF signaling to regulate neuronal function and morphology (Abdelmohsen et al., 2010).

HuR plays a role in adult neurogenesis (Wang et al., 2019) as it is known to translocate to the nucleolus where it functions as a regulator of alternative splicing processes (Nishanth and Jha, 2022). HuR also regulates the focal adhesion kinase (FAK) mRNA which is an essential key player in neurogenesis (Nishanth and Jha, 2022). HuR knockout in transgenic animals leads to a phenotype of neurogenesis and hippocampal-dependent learning in which defects can be observed (Wang et al., 2019). Lack of HuR results in a significant increase in the expression of the FAK isoform with shorter 5′ UTR regions. As a result, FAK function is stimulated which affects neurogenesis. On the other hand, FAK-mediated decreased neurogenesis can be reverted by blocking FAK activity (Bronicki and Jasmin, 2013).

### Lin28

Lin28 is a highly conserved RNA-binding protein encoded by the Lin28 gene (Rehfeld et al., 2015). The Lin28 family includes the isoforms Lin28A and Lin28B, collectively known as Lin28 (Hennchen et al., 2015; Chen et al., 2019). Lin28 is often defined as a pluripotency factor that stimulates cell proliferation, but it also controls other mechanisms such as the timing of cell-time and lineage-specific decisions (Romer-Seibert et al., 2019). The lethal-7 (let7), a key pro-differentiation miRNA, orchestrates posttranslational silencing of mRNAs of neural stem cells (NSC) and acts as an interaction partner with Lin28 (Le Grand et al., 2015; Rehfeld et al., 2015). The members of the let7 family of miRNAs are abundantly expressed in adult tissues, including the brain (Rehfeld et al., 2015), and participate in cell differentiation processes (Hennchen et al., 2015). Lin28 is found in the cytoplasm and cytoplasmic bodies such as processing bodies and stress granules, and partially in the nucleus (Kawahara et al., 2012). While Lin28 is expressed in cell-renewing cells to promote cell proliferation, let7 is absent in stem cells (Rehfeld et al., 2015; Jang et al., 2019). Lin28B can be found in progenitor cells and the cerebral cortex whereas both Lin28A and Lin28B are expressed in neural progenitor cells expressing nestin and Pax6 in the ventricular and SVZ (Hennchen et al., 2015). The expression of Lin28A and Lin28B is not restricted to progenitor cells, but they can also be found in differentiated neuroblasts and expressed at low levels in postmitotic neurons (Hennchen et al., 2015). The expression pattern of Lin28 and let7 reflects the antagonistic interaction between these two players (Rehfeld et al., 2015). During neuron differentiation, let7 expression increases and this overexpression hinders proliferation and stem cell growth mechanisms (Hennchen et al., 2015). This mechanism is implicated in cell proliferation and differentiation of neural stem and precursor cells (Kawahara et al., 2012).

Lin28 contains a unique combination of two coupled and highly conserved functional domains, the N-terminal cold-shock-domain and two retroviral type CCHC-zinc knuckles (CCHCx2) (Rehfeld et al., 2015). Both domains participate in Lin28-mediated posttranscriptional regulation of gene expression as RNA binding takes place in these domains (Rehfeld et al., 2015). Lin28 interacts with the immature let7, also known as pre-let7, which contains the precursor element (PreE) (Rehfeld et al., 2015). The preE has a highly variable sequence structure constituting the loop of the precursor hairpin structure in pre-let7 (Rehfeld et al., 2015). The preE sequence in pre-let7 shows higher sequence variability than the mature let7 form (Rehfeld et al., 2015). Pre-let7 also contains a conserved motif, the GGAG motif, which is highly enriched in let7 family members and is located 3’ to the terminal loop (Rehfeld et al., 2015). The GGAG motif is crucial for the Lin28-mediated inhibition of the maturation of pre-let7 (Rehfeld et al., 2015).

Lin28 participates in several processes such as the generation of induced pluripotent cells from fibroblasts, glucose metabolism regulation, regeneration mechanisms in tissues, body size regulation, progression of cancer, and neurogenesis (Jang et al., 2019). Lin28 acts as one of the regulators participating in the reprogramming of adult cells and the modulatory translational mechanisms of mRNAs, enhancing or suppressing mRNA translation, by binding mRNAs (Parisi et al., 2021). At the early stages of neurogenesis, undifferentiated progenitor cells are present (Hennchen et al., 2015). These cells can be stimulated to proliferate by the action of several regulators. During neurogenesis, the expression of let7 continuously increases until the cell composition is resembling postmitotic neurons (Hennchen et al., 2015). Within the scope of the regulatory mechanisms of neurogenesis, the function of let7 is pivotal because it feeds itself onto the miRNA pathway to prepare the stage for other neurogenic miRNAs in charge of neuronal specifications and outgrowth (Rehfeld et al., 2015).

Lin28-mediated suppression activity of the let7 family promotes cell reprogramming to stimulate pluripotency (Kawahara et al., 2012). Furthermore, Lin28 has demonstrated regulatory effects of the neurite outgrowth process during cortical neurogenesis (Jang et al., 2019). Cell renewal, an event that involves dedifferentiation, is a useful mechanism in tissue regeneration to replace cells that were lost or damaged, and the ability of Lin28 in cell self-renewal could play an important role in this process (Rehfeld et al., 2015). The Lin28-let7 axis in neurogenesis regulates miRNA expression in terms of diversity and abundance during neural differentiation (Rehfeld et al., 2015). Lin28 plays an antagonistic role as it promotes the expression of gene expression patterns specific to stem cells by hindering let7 maturation (Cimadamore et al., 2013). Lin28 suppresses the downstream events regulated by let7 by interfering with the conversion of pre-let7 transcripts to mature let7, which in turn prevents the initiation of the pro-differentiation effect regulated by let7 (Rehfeld et al., 2015; Chen et al., 2019).

Lin28 selectively and strongly binds the conserved terminal loop site of pre-let7 through its specific RNA-binding activity (Kawahara et al., 2012). The interaction of Lin28 with pre-let7 triggers the recruitment of the uridylytransferases Tut4 or Tut7 (Parisi et al., 2021). These transferases catalyze the oligouridylylation of pre-let7 which leads to the degradation of pre-let7 mediated by the exonuclease Dis312 (Parisi et al., 2021). The interaction of Lin28 and let7 forms a self-amplifying system in which cell differentiation is triggered by the lower levels of Lin28 causing less repression of let7 processing and consequently higher levels of let7 and lower levels of Lin28 (Rehfeld et al., 2015; Morgado et al., 2016). Low expression of Lin28 decreases the expression of neuronal markers (Morgado et al., 2016). In the opposite scenario, self-renewal and pluripotency will take place as a result of high levels of Lin28 which cause a reduction in let7 processing resulting in lower levels of let7 and higher levels of Lin28 (Rehfeld et al., 2015; Morgado et al., 2016). The self-reinforcing mechanism of the interaction between Lin28 and let7, a double negative feedback loop, forms a bi-stable switch with two mutually exclusive states, that is, Lin28on-let7off and Lin28off-let7on (Kawahara et al., 2012; Rehfeld et al., 2015). The Lin28-let7 switch mechanism is one of the early events that take place at the onset of neurogenesis (Rehfeld et al., 2015). Lin28 expression significantly decreases during neural stem cell differentiation whereas higher expression of let7 takes place (Morgado et al., 2016). The factors driving the shift between the two interaction states have not been yet identified (Rehfeld et al., 2015).

A challenge in the understanding of the players involved in Lin28-let7 mediated mechanisms is the identification of mRNAs targeted by let7 (Rehfeld et al., 2015). During neurogenesis, let7 is upregulated and can act on important targets such as Lin28, Lin-41, c-Myc, Hmga2, and Tlx (Rehfeld et al., 2015). Some of those interactions participate in stem cell maintenance (Rehfeld et al., 2015) and neurogenesis functions such as cell proliferation (Lin28), axonal regeneration (Lin41), differentiation of Müller glial cells into retinal progenitors and pluripotency networks (c-Myc), stem cell plasticity in the SVZ (Hmga2), and cell cycle progression of NSC (Tlx) (Liu et al., 2014; Rehfeld et al., 2015). During neurogenesis, Lin28 expression is downregulated (Rehfeld et al., 2015).

### Musashi1

Msi1 is a regulator that mediates the balance between self-renewal and cell differentiation (Velasco et al., 2019). Msi1 is expressed in neural stem/progenitor cells of the lateral ventricles, the olfactory subependymal region, and astrocytes in the adult brain (Toda et al., 2001; Takasawa et al., 2002). Low expression levels of Msi1 have been reported in the brain and the expression is limited to the SVZ (Kanemura et al., 2001; Toda et al., 2001), but it can also be found in the subgranular zone of the hippocampus (Ratti et al., 2006). The members of the Musashi family contain two highly conserved tandem RRMs (Toda et al., 2001). Msi1 functions by suppressing the translation of specific mRNA targets, regulating mRNA decay and polyadenylation by binding to transcripts with specific motifs containing ARE at the 3′UTR (Takasawa et al., 2002; Ratti et al., 2006). ARE can be recognized by numerous ARE-binding proteins including the neuronal-specific ELAV (nELAV) RBPs, which are essential to induce neuronal differentiation (Ratti et al., 2006). mRNAs of genes with high turnover rates are known to contain ARE sequences which act as cis-acting regulatory motifs (Ratti et al., 2006). The relationship between Msi1 and nELAV proteins is shown by its co-expression and colocalization with Msi1 in the SVZ (Ratti et al., 2006). Both nELAV and Msi1 act as transcription regulators of Msi1 because of their specificity and ARE-binding activity for the Msi1 transcript (Ratti et al., 2006). nELAV, recognizes the Msi1 transcript in an ARE specific and dependent manner, it stabilizes Msi1 mRNA by decreasing its turnover rate, and this interaction controls proliferation and differentiation activities of neural stem/progenitor cells (Ratti et al., 2006). Therefore, the ARE sequence in the Msi1 gene is involved in mRNA stability and post-translational regulation of Msi1 (Ratti et al., 2006).

nELAV modulates actions of Msi1 at transcript and protein levels, which could alternate the cell cycle by shifting the stem/progenitor cells from cell proliferation to the cell differentiation phase in both neurogenic regions (i.e., SGZ and SVZ). (Ratti et al., 2006). The co-expression of these two proteins in the SVZ is limited to the sub-ependymal cell layer which suggests that they both may be involved in cell proliferation regulatory mechanisms in NSC (Ratti et al., 2006). The functions of Msi1 and nELAV are complementary and act differently on their target mRNAs. For instance, nELAV is responsible for the stabilization of the Msi1 transcript which in turn promotes its expression during the transition from cell proliferation to cell differentiation (Ratti et al., 2006). Overexpression of Msi1 is an indicator of the proliferation state (Ratti et al., 2006). Therefore, transcription and translational mechanisms regulating Msi1 expression will have an effect on Msi1 levels during the cell proliferation phases (Ratti et al., 2006). Msi1 is highly expressed during neurogenesis (Pötschke et al., 2020). nELAV RBPs are expressed in the SVZ and colocalize with Msi1 in neural stem/progenitor cells (Ratti et al., 2006). The inhibitory effect of Msi1 on cell differentiation is observed in neurogenesis. On the other hand, Msi1 is upregulated in NPCs, which supports its role in cell proliferation (Pötschke et al., 2020).

Msi1 expression during neurogenesis goes in the opposite direction to the expression levels of miR-137 (Velasco et al., 2019). In the SVZ, expression levels of Msi1 and miR-137 are inversely correlated (Velasco et al., 2019). miR-137 decreases self-renewal and cell proliferation while Mis1 increases cell proliferation (Velasco et al., 2019). Msi1 expression is higher in the SVZ where it promotes self-renewal and proliferation of stem cells while the expression of miR-137 is also high as it is required for lineage progression and cell differentiation (Velasco et al., 2019). miR-137 drives cell differentiation and inhibits Msi1 by suppressing the expression of shared targets (Velasco et al., 2019). miR-137 shares targets with miR-124 and miR-128, from which miR-128 is also known to regulate Msi1, and they all work in a synergistic fashion regulating neurogenesis (Velasco et al., 2019).

Msi1 acts on the Notch-mediated proliferation pathway in NSC where it binds and prevents the translation of Numb resulting in inhibition of Notch activation (Ratti et al., 2006; Pötschke et al., 2020). Msi1 participates in the downregulation of several regulators such as Numb, a negative regulator of Notch; p21, an inhibitor of cyclin-dependent kinases, and doublecortin, a microtubule-binding protein involved in cell migration (Glazer et al., 2012). Conversely, Mis1 promotes the upregulation of Rondabaout3 (Robo3), a receptor involved in axonal guidance (Glazer et al., 2012).

Another mediator of Msi1 activity is HuD which participates in the stabilization of Msi1 mRNA to promote Msi1-mediated cell proliferation of NPCs (Ratti et al., 2006; Pötschke et al., 2020). This mechanism allows the NPCs to keep dividing disregarding the transcriptional inactivation of Mis1 (Ratti et al., 2006). HuR positively regulates Msi1 expression (Velasco et al., 2019). Msi1 also interacts with Lin-7 by stimulating the inhibitory effect of Lin28 on let7 during cell differentiation (Lang and Shi, 2012).

### Sam68

Sam68, the Src-associated substrate during mitosis of 68 kDa, also known as the human KH domain containing RNA binding signal transduction associated 1 (KHDRBS1), is a member of the Signal Transduction Activator of RNA (STAR) family of RBPs (Lim et al., 2006; Vogel and Richard, 2012; Danilenko et al., 2017). The proteins of the STAR family are highly conserved and participate in cell proliferation and cell differentiation processes (Bielli et al., 2011). Sam68 is mainly found in the nucleus, but it can also be expressed in the soma and dendrites of neurons in the hippocampus, cortex, and the SVZ (Lim et al., 2006; Chawla et al., 2009). Structurally, Sam68 has a 96-amino acid sequence at the N-terminus followed by a STAR domain, which contains a KH domain and flanking regions involved in protein-protein and protein-RNA interactions, an arginine-glycine rich domain, and a tyrosine-proline rich domain at the C-terminus (Huot et al., 2009; Danilenko et al., 2017). The arginine-glycine and tyrosine-proline domains are prone to posttranslational modifications (Danilenko et al., 2017). The highly conserved N- and C- terminal sequences are essential for RNA binding and homodimerization, and they are harbored by a single KH domain (Vogel and Richard, 2012).

Sam68 plays a role in RNA metabolism including polysomal recruitment of mRNAs and alternative splicing (Vogel and Richard, 2012). Sam68 regulates alternative splicing through the recognition of RNA sequences rich in adenine and uracil neighboring the included/excluded exons (Vogel and Richard, 2012). Sam68 can be subjected to post-translational modifications such as phosphorylation (serine/threonine and tyrosine), acetylation (lysine), methylation (arginine), and sumoylation (arginine) that regulate its subcellular localization, interactions with signaling proteins, and affinity for target RNAs (Bielli et al., 2011; Vogel and Richard, 2012). For instance, the proline and tyrosine-rich regions make Sam68 a substrate of many kinases such as the Scr family kinases, phospholipase Cγ1, Grb2, Nck, and Csk (Huot et al., 2009). The tyrosine phosphorylation of Sam68 by Scr-related kinases greatly affects its ability to form homodimers and interact with target RNAs in the cell (Bielli et al., 2011). The arginine-rich region of Sam68 undergoes methylation by the methyltransferase PRMT1 (Bielli et al., 2011). The nuclear translocalization of newly formed Sam68 is affected by the arginine methyltransferase PRMT1 mediated methylation of Sam68, which hinders the interaction of Sam68 with SH domains (Bielli et al., 2011). Arginine methylation of Sam68 is a prerequisite for its successful nuclear localization (Côté et al., 2003).

Sam68 participates in the alternative splicing of mRNAs involved in neurogenesis (Huot et al., 2009). In neuronal cells, a set of 24 novel exons regulated by Sam68-mediated splicing had been identified and associated with neurogenesis (Chawla et al., 2009). Genes carrying Sam68 targets exons act in processes involved in neurogenesis such as cytoskeletal organization (Numa1, Clasp2, and Sgce), biogenesis, and transport of organelles (Bin1, Km1, Kifap3, and Opa1) and synaptogenesis (Cadm1, Dlgh4, and Sorbs1) (Chawla et al., 2009). Furthermore, Sam68 mediated the maintenance of splicing patterns needed after cell differentiation (Vogel and Richard, 2012). Sam68 is one of the 11 splicing factors highly expressed in the SVZ and the olfactory bulb core implicated in adult SVZ neurogenesis (Lim et al., 2006). Neuroblasts born in the SVZ can either integrate into the granule cell layer or migrate to the periglomerular layer in the olfactory bulb (Lim et al., 2006). Because alternative splicing allows the cells to regulate the same set of transcription factors that can affect the generation of different phenotypes of newborn neuroblasts in the SVZ, this mechanism may determine neuroblast migration or the cell fate choice (Lim et al., 2006). In the olfactory bulb core, Sam68 changes its subcellular localization, interaction with the spliceosome, and splice site selection upon phosphorylation by the kinase Fyn (Hartmann et al., 1999). Fyn overexpression in the olfactory bulb leads to changes in the Sam68-mediated mRNA splicing in type A cells resulting in cell cycle exit, radial migration, and integration of cells into local circuits (Lim et al., 2006). Furthermore, Sam68 associates the SVZ precursor RNA splicing machinery with the extracellular environment (Lim et al., 2006). For instance, the splicing activity of Sam68 in the SVZ is regulated by the extracellular signal-regulated kinase (ERK) (Lim et al., 2006).

### Staufen 1

Stau1 and Stau2 are involved in RNA transport as well as mRNA stability and translation (Almasi and Jasmin, 2021). The Stau1 gene encodes the Stau1 protein (Bondy-Chorney et al., 2016). The five alternative splice variants produced by mature Stau1 mRNAs differ in their 5′UTR regions (Almasi and Jasmin, 2021). While Stau2 is abundant in the brain and only weakly expressed in other tissues, Stau1 is present in the majority of cell types, including neurons (Duchaîne et al., 2002). In mature hippocampus neurons, Stau1 is known to localize mRNA. The two proteins are mostly present in separate particles in the dendrites of hippocampal neurons, which may indicate that they have different roles (Duchaîne et al., 2002). Stau1 is also preset in the SVZ (Moon et al., 2018).There are two major kinds of Stau1 binding sites. Pairs of Alu elements in 3′ UTRs are included in the first class while non-Alu sequences are the second kind of Stau1-binding site (Almasi and Jasmin, 2021). Several target mRNAs have been found to include non-Alu 3′UTR binding sites (Almasi and Jasmin, 2021).

Stau1 and Stau2 are crucial for the transport and localization of certain mRNAs into the dendrites of adult hippocampal neurons and their knockdown in these cells impairs synaptic plasticity (Goetze et al., 2006; Vessey et al., 2008). Stau1 is involved in cell growth (Ghram et al., 2020), differentiation (Gautrey et al., 2005), migration, apoptosis (Gandelman et al., 2020), autophagy (Paul et al., 2021), and the stress response (Thomas et al., 2009; Bondy-Chorney et al., 2016; Paul et al., 2021). Stau1 is crucial for NPCs’ development since over-expressing Stau1 in NPC cultures improves the detection of neuron-specific genes (Crawford Parks et al., 2017). In NPCs, Stau1 moves back and forth between the cytoplasm and nucleus (Kusek et al., 2012; Vessey et al., 2012). Stau1 can proceed *via* nuclear import and export in NPCs. Stau2 plays a critical role in the determination of cell fate during neurogenesis (Kusek et al., 2012; Vessey et al., 2012). Stau1 regulates neurogenesis by interacting with mRNA targets such as Dlx1, Dlx2, and Tuj1 (Moon et al., 2018). Stau1 acts by promoting the degradation of Dlx1, Dlx2, and Tuj1 mRNAs while knocking down Stau1 results in the inhibition of the degradation of target mRNAs suggesting that Stau1 is involved in the stability of those target mRNAs associated with neurogenesis (Moon et al., 2018).

### SRY (sex-determining region)-box 2

The *Sry* (Sex-determining Region Y) gene was found as an initial member of the SOX gene family capable of determining the male phenotype (Gubbay et al., 1990). While members of the SOX family share similar DNA-binding properties, individual SOX proteins bind specific partner proteins to regulate their target genes (Kamachi et al., 2000; Tanaka et al., 2004). The SOX genes are divided into subgroups B1 (SOX1, SOX2, and SOX3) and B2 (SOX14 and SOX21) (Uchikawa et al., 1999). The SOX family of proteins contains a domain of 79 amino acids that allows them to bind specifically to the sequence (A/T A/T CAA A/T) (Harley et al., 1994) and two domains that function in transcriptional regulation (Pevny and Lovell-Badge, 1997). The SOX proteins bind to the minor groove and, upon binding, induce strong bends in DNA (Michael, 1999).

Two types of NPCs express SOX2, the quiescent radial NPCs (type 1) and the amplifying progenitors (type 2a) (Ellis et al., 2004; Ferri et al., 2004; Garcia et al., 2004; Seri et al., 2004; Suh et al., 2007). Throughout life, SOX2 is expressed in the developing hippocampus, cortical hem (CH), and dentate neural epithelium (DNE), and then continues to be expressed in the dentate gyrus (Mercurio et al., 2022). Compared to surrounding tissues, SOX2 expression is significantly enriched in the CH (Ferri et al., 2004; Favaro et al., 2009; Mercurio et al., 2022), suggesting a critical role for SOX2 in this area. NPCs express SOX2 before turning it off in differentiated neurons (Hodge and Hevner, 2011). SOX2 expression decreases during differentiation when progenitor cells become postmitotic during their final cell cycle (Graham et al., 2003). A cell expressing SOX2 is capable of producing both identical cells and differentiated neural cells, two hallmarks of stem cells (Liu et al., 2019).

SOX2 is a well-known regulator of cell proliferation and neurogenesis, and it participates in the upstreaming events of the Lin28-let7 axis (Mukherjee et al., 2011; Rehfeld et al., 2015). In adult NSC in the subgranular zone of the hippocampus and the SVZ, Lin28 expression correlates with increased SOX2 activity (Rehfeld et al., 2015). SOX2 binds to the Lin28 promoter triggering the recruitment of histone deacetylase complexes which upregulates Lin28 expression (Rehfeld et al., 2015). The loss in neurogenesis caused by depletion of SOX2 can be partially compensated by overexpression of Lin28 by interfering with the functional maturation of let7 (Cimadamore et al., 2013; Rehfeld et al., 2015; Morgado et al., 2016). Another event demonstrating the cross-talk interaction of SOX2 and the Lin28-let7 axis in neurogenesis is that let7 downregulates the neurogenic basic-helix-loop-helix transcription factors Ascl1/Mash1 and neurogenin (Rehfeld et al., 2015). These series of events suggest that suppression of let7 expression by maintaining Lin28 expression is a requirement, at least in part, for SOX2 in neurogenesis (Rehfeld et al., 2015).

## Discussion

In this review, RBPs involved in the different stages of the adult neurogenesis process were discussed. RBPs such as Sam68 and Msi1 are involved at all stages of neurogenesis (Figure 1). The regulatory specificity of Sam68 and Msi1 in neurogenesis may be driven by the interaction of these RBPs with different target mRNAs. The role of specific mRNA partnerships at each stage of neurogenesis suggests the possibility to manipulate a particular stage *via* a specific regulatory partnership. However, it can also be observed that the same RBP-mRNA partners (e.g., Msi1-miR137, Sam68-Fyn, HuR-Fak, Lin28-let7) also have the ability to act on different neurogenesis phases through the same mRNA targets. This phenomenon invites to ask whether other regulatory mechanisms are involved and whether there is a cross-talk among RBPs contributing to the regulation at each stage of the neurogenesis process. A clear case of an RBP-RBP cross-talk mechanism was shown in SOX2 in which this RBP interacts with the Lin28-let7 axis. Some RBPs such as HuD, Sam68, Msi1, and Stau1 act on multiple mRNA targets to regulate a specific stage of the neurogenesis process which raises the question whether there could be more target mRNAs that have not yet been identified for the other RBPs. At times, the process to activate or deactivate RBP mediated function has been clearly identified such as in Lin28 case, but often times the activation-deactivation process is not well understood. What is clear from the analysis presented in this review is that there is an intricate RBP-mediated regulatory network taking place in neurogenesis. Tracing the regulatory network mediated by RBPs in neurogenesis will potentially contribute to understanding the impact and clinical implications on cognition and mood. Therefore, efforts should be made to clarify the RBP-mediated regulatory mechanisms and to identify relevant mRNAs involved in neurogenesis.

## Conclusion

Gene expression mechanisms, including the control of mRNA synthesis *via* RBPs, are backbone mechanisms that shape the architecture of the CNS. Several RBPs are involved in the intricate regulation of neurogenesis at all stages from, cell proliferation, migration, and differentiation, to integration into the existing circuit in both the SVZ and the dentate gyrus of the hippocampus (Ming and Song, 2011).

It is important to note that identifying potential therapeutic targets to modulate neurogenesis at a genetic level demands our understanding of RBPs considering 1) the bidirectional regulation of neurogenesis (e.g., agonistic and antagonistic regulatory effect on neurogenesis), 2) self-amplification effect (e.g., Lin28), 3) overlapping RBPs targets and effectors (e.g., Lin28 and Msi1, miR-145 mediating the downregulation of SOX2 and Lin28), and 4) complementary actions among RBPs (e.g., Msi1 and nELAV) as discussed in the review. Furthermore, the degree of understanding of the RBP mechanism, their molecular regulatory partners, and neurogenic region of action has been slowly emerging being HuD, HuR, FXR2, Lin28, Msi1, and Sam68 the most characterized RBPs to date. Despite the advances to understand the complex RBP-mediated regulation of neurogenesis, more research is needed to trace a clear map of the molecular regulatory mechanisms and the critical players to identify potential therapeutic neurogenic promoting targets.
